# Closing the gap: proposing a socio-ecological framework to make cancer clinical trials more accessible, equitable, and acceptable to adolescents and young adults

**DOI:** 10.1093/oncolo/oyae257

**Published:** 2024-09-27

**Authors:** Zeba Ahmad, Vinayak Venkataraman, Michaela Markwart, Annah N Abrams, Jennifer S Temel, Giselle K Perez

**Affiliations:** Health Promotion and Resiliency Intervention Research Center (HPRIR), Massachusetts General Hospital, Boston, MA 02114, United States; Harvard Medical School, Boston, MA 02115, United States; Center for Psychiatric Oncology and Behavioral Sciences, Massachusetts General Hospital, Boston, MA 02114, United States; Harvard Medical School, Boston, MA 02115, United States; Department of Medical Oncology, Dana-Farber Cancer Institute, Boston, MA 02215, United States; Health Promotion and Resiliency Intervention Research Center (HPRIR), Massachusetts General Hospital, Boston, MA 02114, United States; Harvard Medical School, Boston, MA 02115, United States; Center for Psychiatric Oncology and Behavioral Sciences, Massachusetts General Hospital, Boston, MA 02114, United States; Division of Child and Adolescent Psychiatry, Mass General for Children, Boston, MA 02114, United States; Division of Pediatric Hematology/Oncology, Mass General for Children, Boston, MA 02114, United States; Harvard Medical School, Boston, MA 02115, United States; Division of Hematology/Oncology, Mass General Cancer Center, Boston, MA 02114, United States; Health Promotion and Resiliency Intervention Research Center (HPRIR), Massachusetts General Hospital, Boston, MA 02114, United States; Harvard Medical School, Boston, MA 02115, United States; Center for Psychiatric Oncology and Behavioral Sciences, Massachusetts General Hospital, Boston, MA 02114, United States

**Keywords:** AYA, adolescent and young adults, clinical trials, rare cancers, psychosocial research

## Abstract

This commentary focuses on what would make cancer clinical trials more accessible, equitable, and acceptable to adolescents and young adults.

## Optimizing AYA CCT participation with a socio-ecological approach

Each year, 85 000 adolescents and young adults (AYAs, 15-39 years old) are diagnosed with cancer in the United States.^1^ Despite advances in treatment, AYAs have lower 5-year survival rates than younger and older patients.^2^ Treatments and techniques are developed through cancer clinical trials (CCTs), which are vital to combatting cancer and improving survivorship. National guidelines strongly encourage CCT participation for all eligible AYAs.^3^ However, persistent barriers to participation include compounded disparities for AYAs based on their ethnic/racial identity, socioeconomic background, cancer type, treatment setting, and location.^4^ Limited and inequitable participation in CCTs hinders the scientific understanding of tumor biology, treatment responses, toxicities, and the development of novel therapies—perpetuating the survival gap.^5^

**Figure 1.**
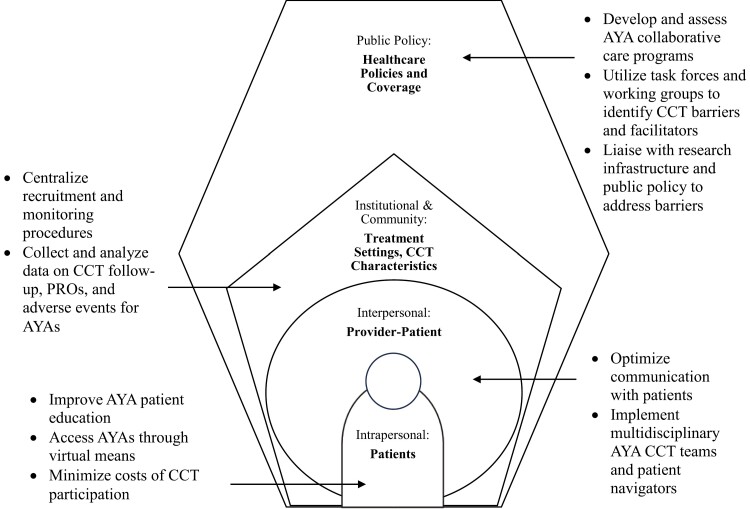
Socio-ecological framework for suggested interventions to optimize adolescent & young adult enrollment in cancer clinical trials.

Here, we summarize barriers and facilitators and suggest strategies that would make CCTs more accessible, equitable, and acceptable to AYAs. Our recommendations are guided by the socio-ecological model (SEM, Figure 1),^6^ which proposes multiple, reciprocal levels of influence on behavior: intrapersonal (ie, patient), interpersonal (patient-provider), institutional and community (treatment setting and CCT characteristics), and public policy (health policy and insurance). The SEM is apt because CCT participation encompasses the multistep process of identifying, recruiting, enrolling, treating, and monitoring patients; therefore, barriers and disparities are multifactorial. We propose that the gaps in AYA CCT participation reflect opportunities to optimize several levels of care for this vulnerable population.

## Socio-ecological barriers, facilitators, and strategies for AYA participation in CCTs

### Intrapersonal: patient-level

Cancer clinical trial (CCT) participation includes identification of appropriate trials, enrollment, and follow-up monitoring; access may be most limited when care disparities coincide. Racial and ethnic marginalization intersect with socioeconomic factors such as income and location to contribute to vulnerabilities in care. For example, AYAs in SWOG clinical trials are less racially/ethnically diverse than the AYA cancer population. Black AYAs are less likely than White AYAs and Black pediatric patients to participate,^5^ and Black males are less likely to participate than females, with male enrollment rates reducing at higher AYA age.^7^ Another study estimated that individuals who were Hispanic and/or insured through public policies (eg, Medicaid) were least likely to join CCTs.^8^ AYAs overall may be twice as likely to be lost to follow-up compared to pediatric patients, and Black patients, individuals with germ cell tumors, and patients estimated to have a median household income below the poverty line are at the highest risk.^9^

Qualitative research provides insight into the attitudes and experiences of AYAs considering CCTs. Concerns include losing autonomy and feeling like a “guinea pig” in the context of medical mistrust, not understanding the benefits of trials, information overload, cryptic medical jargon, and the perception of eligibility criteria as unclear or exclusionary. Worries about safety and comprehension may be highest among individuals with English as a second language and those who have most recently received their cancer diagnosis.^10,11^ AYAs describe CCTs as less safe and more burdensome than older adults do.^12^ Due to both direct and indirect participation costs,^13^ AYAs may perceive CCTs as costly in time and resources when they are already missing out on normative developmental milestones.^10^ Expenses of childcare and education/professional training can intersect with socioeconomic vulnerabilities, including inconsistent insurance and lower economic security, to hinder participation.

Patient-facing facilitators of participation include utilizing social networking strategies (eg, Internet-based groups) for recruitment, and monetary incentives and email to improve retention.^14^ AYAs who participate in collaborative CCT decision-making report greater autonomy, research engagement, and treatment adherence.^10^ AYA support groups and mentorship help cultivate peer relationships, which bolster coping and self-efficacy.^15^ Care teams should facilitate CCT participation by offering patient education programs with developmentally appropriate materials that inform patients and families about the importance and benefits of CCTs, dispel myths and misconceptions, and share the benefits of participation to advance knowledge and treatment. Such materials can be developed and refined on a centralized virtual platform.

### Patient-provider level

Barriers expressed by oncology providers include limited awareness of appropriate CCTs, administrative and logistical concerns, and inconsistent care coordination between pediatric and medical oncology teams. Providers report less experience caring for AYAs and their rarer cancers, and hesitancy in delaying the start of treatment and due to potential exposure to irreversible complications (eg, loss of fertility).^10,11,16^

Improving communication and mutual understanding between patients and providers regarding CCTs, and coordination of care related to CCTs, would respond to these limitations.

#### Communication

To improve research engagement, clinicians must understand how their patients’ developmental stage impacts their attitudes, informational needs, decisional capacity, and preferences. Members of medical oncology teams can seek consultation from AYA specialists or clinical trialists regarding best practices to broach CCT discussions and involve AYAs’ families in the decision-making process. Research should determine the optimal communication style (tone and pace), mode (in-person and virtual), and supplementary tools (handouts, websites, and social media) to share CCT information and make the best use of constrained time during medical visits. Education and communication interventions can be designed to empower AYAs to make complex decisions.

#### Coordinated care and patient navigators

Interventions to improve CCT participation should involve multidisciplinary provider teams, including oncologists, nurses, allied health professionals, psychiatrists, psychologists, social workers, health services researchers, implementation scientists, patient advocates, clinical trialists, and patient navigators. Collaborative care programs should offer training for clinicians and staff to help them understand and share information about CCTs with AYAs.

Literature supports the implementation of patient navigators, who educate, identify needs, and overcome barriers to care among underserved populations.^17-19^ AYAs describe nurse navigators as helpful in answering questions, navigating logistical and financial hurdles, and providing impartial advice.^11^ Lessons learned from interventions in adult and underserved populations include conducting root cause analyses of low participation, ensuring culturally tailored communication, and considering the role of loved ones,^17,20^ which can help design AYA-specific navigation programs.

### Community-level: treatment setting and CCT characteristics

Compared to that of National Cancer Institute-designated (NCI) Comprehensive Cancer Centers and academic medical centers, significantly lower rates of CCT participation are reported among patients treated in community health centers and in rural areas. Pediatric cancer centers offer CCTs that may not be available to same-aged AYAs receiving care at affiliated adult cancer sites.^10,11,16^ Specific barriers to CCT participation include the site of oncologic care, distance to tertiary facilities, CCT eligibility criteria (eg, follow-up monitoring requirements), and social determinants of health (eg, healthcare costs and transportation).^21^ Opening and maintaining under-enrolled CCTs can be costly for cancer treatment sites, which therefore may elect to prioritize funding for the most prevalent types of cancer or commonly affected age groups; this cost-effectiveness concern may limit opportunities for AYAs to obtain care and slow the development of novel treatments. Systemic facilitators of enrollment include effective care coordination between medical and pediatric oncology teams, an infrastructure that supports research, and the involvement of AYA clinical champions such as in specialized AYA programs.

#### Collect data most relevant to AYA CCTs

To better address ethnic/racial and socioeconomic disparities in which patients are recruited to CCTs, and which patients are lost to follow-up, enhanced data monitoring and identification of benchmarks are needed. Within CCT data, reporting of adverse event data in AYAs is not distinguished from older adults; this is key information for patient care and treatment advancement.^22^ Additionally, we advise further integration of patient-reported outcomes (PROs) into CCT benchmarks. Barriers include age-appropriate PRO measures, fees, and language; similar to the PROMIS initiative, we recommend centralizing measurement practices and making them available online.

### Society-level: collaborative care, insurance coverage, public policy, and community-based organizations

AYA enrollment in CCTs remains low, but upward trends over the past 25 years may reflect the expansion of healthcare coverage to dependents aged below 26, and benefit from NCI and collaborative group initiatives to improve CCT enrollment. A coordinated care model that systematically addresses AYA-specific challenges is needed. This includes creating systems to facilitate coordination between AYA specialists and clinical trialists, enable developmentally appropriate communication with patients, improve the caliber of follow-up care and data collection, and establish synchronous and asynchronous outreach initiatives to address obstacles and promote engagement with CCTs.

#### Leverage collaborative care opportunities and centralize resources

Coordinated efforts can improve targeted age-concordant care and CCT access while addressing gaps in a fragmented healthcare system. Tailoring is required, as treatment centers have distinct challenges: for example, clinics with fewer AYA enrollments report difficulty locating open CCTs, and clinics with higher enrollment report limited support from physicians.^23^ Effective initiatives may take the form of AYA programs,^24^ regional research and clinical collaboratives, working groups from nationwide cancer care programs, and innovative task forces. For example, pooling clinical trials across oncological treatment sites could improve enrollment in CCTs and make them less costly for each individual site. These programs and models should be further developed to extend their reach and monitored regularly for sustainability and effectiveness.

For instance, the AYA oncology program at the University of Pittsburgh Medical Center and Children’s Hospital of Pittsburgh leveraged their existing research network and infrastructure to augment the CCTs available to AYAs. They disseminated CCT updates at regular meetings between pediatric and adult stakeholders.^25^ The AYA Trial Access Quality Initiative took a multidisciplinary approach to quality improvement to reduce barriers to CCT enrollment, including problem-solving about tracking AYA recruitment data and learning sessions with clinical experts about oncology care coordination, with plans to incorporate stakeholder input.^26^ Using a toolkit made available online, a series of working groups in Australia took a coordinated approach to improve protocol approval, awareness, access to pediatric hospitals, and streamlining enrollment procedures across institutions and by collaborating with governmental officials.^27^ In order to improve reach, access, and enrollment to CCTs, a statement from the Children’s Oncology Group AYA Oncology Discipline Committee advises using remote visits for screening, recruitment and consent, and follow-up care.^28^

We suggest further collaboration between specialized treatment sites, such as AYA programs at academic medical centers, and community treatment settings. Conducting enrollment virtually, and monitoring (eg, lab and imaging procedures) locally, is a powerful combination to address disparities in access. We further suggest that collaborative care programs can involve information technology to establish a cancer care summary template to share between cancer care teams during interdisciplinary meetings and integrate information about CCTs within the EHR to facilitate discussion and streamline referrals. Clinical programs may partner with AYA advisory boards and patient advocacy groups to amplify their efforts to promote CCT awareness, education, and participation. These efforts could culminate in the development of consultation programs for CCT, wherein investigators can solicit input for strategies to reduce barriers to participation and boost AYA engagement in CCTs.

To offset the costs of opening and maintaining CCTs for AYAs, collaborative care programs may seek grants and philanthropic funding from community-based organizations (CBOs) that advocate for AYA care (ie, Teen Cancer America); collaborative care programs and CBOs may collectively appeal to pharmaceutical companies conducting clinical trials to subsidize costs for sites, improving feasibility. Hospital-based programs can also advocate for the placement of stakeholders on IRBs to address common procedural and structural challenges that disproportionally affect AYAs.

## Conclusion

AYAs face cancer and complex medical decisions during a critical developmental period and in the context of health disparities in care access; efforts to encourage trial participation need to be tailored accordingly. By optimizing CCT participation facilitators with a socio-ecological framework, progress can be made to improve access and acceptability of CCTs for AYAs.

## Data Availability

No new data were generated or analysed in support of this research.
